# The Database for Aggregate Analysis of ClinicalTrials.gov (AACT) and Subsequent Regrouping by Clinical Specialty

**DOI:** 10.1371/journal.pone.0033677

**Published:** 2012-03-16

**Authors:** Asba Tasneem, Laura Aberle, Hari Ananth, Swati Chakraborty, Karen Chiswell, Brian J. McCourt, Ricardo Pietrobon

**Affiliations:** 1 Duke Clinical Research Institute, Durham, North Carolina, United States of America; 2 Department of Surgery, Duke University School of Medicine, Durham, North Carolina, United States of America; University of Michigan, United States of America

## Abstract

**Background:**

The ClinicalTrials.gov registry provides information regarding characteristics of past, current, and planned clinical studies to patients, clinicians, and researchers; in addition, registry data are available for bulk download. However, issues related to data structure, nomenclature, and changes in data collection over time present challenges to the aggregate analysis and interpretation of these data in general and to the analysis of trials according to clinical specialty in particular. Improving usability of these data could enhance the utility of ClinicalTrials.gov as a research resource.

**Methods/Principal Results:**

The purpose of our project was twofold. First, we sought to extend the usability of ClinicalTrials.gov for research purposes by developing a database for aggregate analysis of ClinicalTrials.gov (AACT) that contains data from the 96,346 clinical trials registered as of September 27, 2010. Second, we developed and validated a methodology for annotating studies by clinical specialty, using a custom taxonomy employing Medical Subject Heading (MeSH) terms applied by an NLM algorithm, as well as MeSH terms and other disease condition terms provided by study sponsors. Clinical specialists reviewed and annotated MeSH and non-MeSH disease condition terms, and an algorithm was created to classify studies into clinical specialties based on both MeSH and non-MeSH annotations. False positives and false negatives were evaluated by comparing algorithmic classification with manual classification for three specialties.

**Conclusions/Significance:**

The resulting AACT database features study design attributes parsed into discrete fields, integrated metadata, and an integrated MeSH thesaurus, and is available for download as Oracle extracts (.dmp file and text format). This publicly-accessible dataset will facilitate analysis of studies and permit detailed characterization and analysis of the U.S. clinical trials enterprise as a whole. In addition, the methodology we present for creating specialty datasets may facilitate other efforts to analyze studies by specialty groups.

## Introduction

ClinicalTrials.gov (www.ClinicalTrials.gov) is a registry of human clinical research studies. It is hosted by the National Library of Medicine (NLM) at the National Institutes of Health (NIH) in collaboration with the U.S. Food and Drug Administration (FDA). As mandated by federal law [1], ClinicalTrials.gov provides a central resource for information about clinical trials; in addition, it increases the public visibility of such research. The registry currently contains over 100,000 research studies conducted in more than 170 countries and is widely used both by medical professionals and the public. New research studies are being submitted to the registry by their respective sponsors (or sponsors' designees) at a rate of approximately 350 per week [2]. Due to legislative [1] and institutional [3] requirements enacted in the latter half of the previous decade, compliance with registry obligations is assumed to be high for U.S. drug and device trials, and the consistency, quality, and maintenance of registry data have improved with increased use [4]. However, the registry has not been optimized for the analysis of aggregate data, and a systematic effort to create and maintain a database for this purpose has not previously been undertaken.

In November 2007, the FDA and Duke University announced the formation of a public-private partnership to improve the quality and efficiency of clinical trials. This collaboration of more than 60 organizations and government agencies was convened by Duke University under a memorandum of understanding with FDA, and is now known as the Clinical Trials Transformation Initiative (CTTI) [5]. CTTI leaders recognized that ClinicalTrials.gov represented a promising source for benchmarking the state of the clinical trials enterprise, as the registry contains studies from the full range of sponsoring organizations. Increasing the usability of ClinicalTrials.gov data may therefore facilitate systematic evaluation of clinical studies aimed at building the knowledge base needed to inform medical practice and prevention.

As data have accumulated in ClinicalTrials.gov, users have increasingly sought capabilities that would allow aggregated descriptive characterization of the national research portfolio; however, access and data usability issues, including data format and design, present obstacles. A number of related initiatives, including the Ontology of Clinical Research (OCRe) [6], Human Studies Database (HSDB) [7], CDISC Protocol Representation Model [8], and LinkedCT [9] projects, are addressing ontological annotations, large-scale data mining, data representation format, and external association of these data, respectively. The results of this project are complementary to these initiatives and are expected to collectively advance this area of study as a whole.

In this article, we report on CTTI's efforts to prepare and maintain a publicly accessible analysis dataset derived from ClinicalTrials.gov content—the database for aggregate analysis of ClinicalTrials.gov (AACT). We also discuss efforts to extend the utility of the analysis dataset by means of an associated clinical specialty taxonomy designed to support research policy analyses.

## Methods

### 1. Creation of the AACT

**Figure 1:**
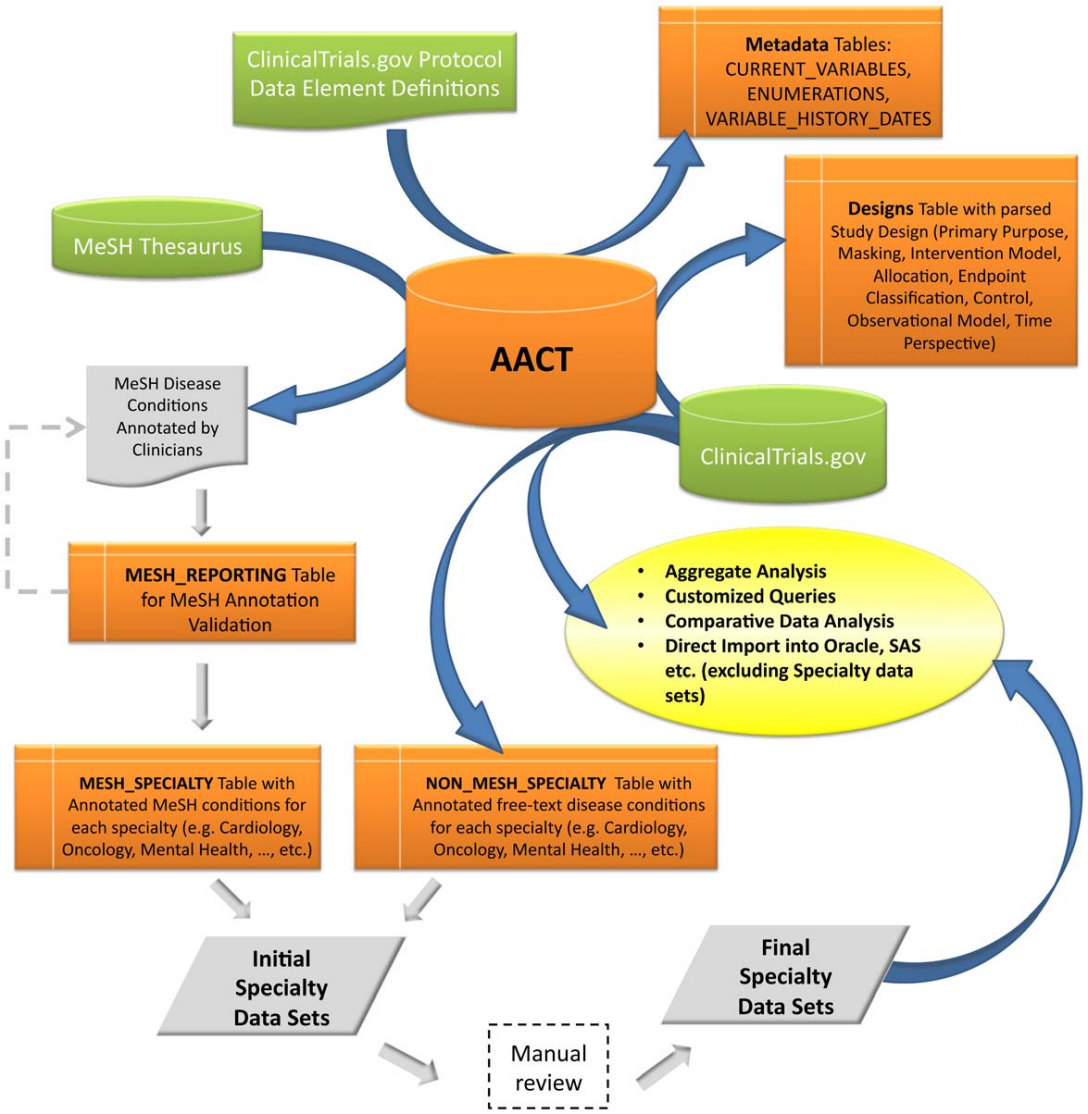
A schematic representation of the database for Aggregate Analysis of ClinicalTrials.Gov (AACT) with its key enhancements.

Key design features of AACT include 1) the capacity to extend the dataset by parsing existing data; 2) linking to additional data resources, such as the Medical Subject Headings (MeSH) thesaurus; and 3) integrated metadata. A framework for extensions allows entire studies or individual fields to be associated with new data resources while preserving provenance. In addition, the integrated data dictionary developed for this project facilitates browsing and analysis of ClinicalTrials.gov and AACT metadata. Finally, the database incorporates a flexible design that can accommodate future developments, such as coding biospecimen type, sponsors, and OCRe annotations. Figure 1 shows key enhancements achieved by building the AACT.

#### 1.1. Data Sources

A dataset comprising 96,346 clinical studies was downloaded from ClinicalTrials.gov in XML format on September 27, 2010. We chose ClinicalTrials.gov for our study because it is the largest database of its kind and because it covers the full range of clinical conditions, includes a broad group of trial sponsors [10], and has a regulatory mandate [1]. The date of download was chosen to coincide with the anniversary of the enactment of the FDA Amendments Act (FDAAA) 3 years earlier, which mandated the registration of certain trials of FDA-regulated drugs, biologics, and devices [1].

We downloaded the 2010 MeSH thesaurus (http://www.nlm.nih.gov/mesh/2010/download/termscon.html) and merged it with the AACT database, where it was used as a lookup table to locate corresponding tree numbers, referred to as *MeSH IDs*, for all MeSH terms associated with each clinical trial in ClinicalTrials.gov. Persons or organizations who submit studies to the registry are requested to provide the *condition* and *keyword* data elements as MeSH terms.

#### 1.2. Data Model

**Figure 2:**
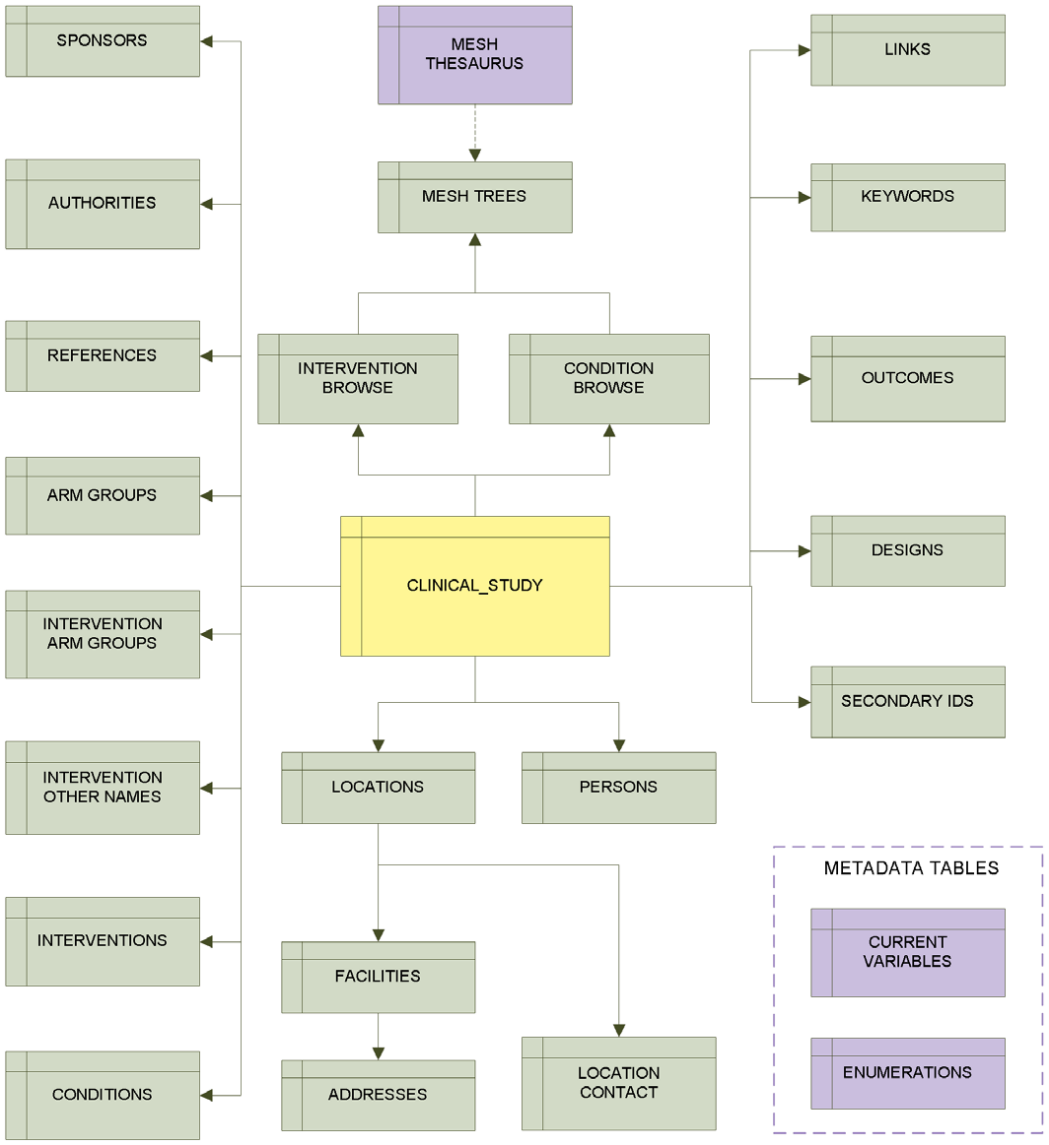
High-level Entity-Relationship Diagram (ERD) for AACT.

ClinicalTrials.gov data element definitions, xsd specifications for registry data submission, and downloaded study XML files were used to represent data specifications for the downloaded data. A physical data model was designed using Enterprise Architect (Sparx Systems Pty Ltd, Creswick, Victoria, Australia); this model depicted data tables and their data columns, as well as relationships between and among tables. An optimal structure was achieved through normalization, which was used to organize data efficiently, eliminate redundancy, and ensure logical data dependencies by storing only related data within a given table [11]. The database (Figure 2) was normalized to the Second Normal Form (2NF), a set of criteria designed to prevent logical inconsistencies while reducing data redundancy [12].

We assigned data type and length of data elements based on patterns observed for each data element in the downloaded XML files. Whenever possible, we followed guidelines provided in ClinicalTrials.gov's draft Protocol Data Element Definitions [13] when assigning lengths to given data elements. Data were housed in Oracle RDBMS, version 11.1 g (Oracle Corporation, Redwood Shores, California, USA). Enterprise Architect 7.1 was used for database design and additional transformation rules were documented as extract-transform-load (ETL) specifications. PL/SQL packages that used Oracle's inbuilt DBMS_LOB package to read the input XML files and load the data into the designed tables appropriately were developed. Quality control and operational support processes were developed using standard SQL queries through Toad for Data Analysts (Quest Software, Aliso Viejo, CA, USA) and Cognos ReportNet (CRN) (IBM Corporation, Armonk, NY, USA). We extended the core data model to accommodate both data management and data curation purposes. Error log tables and indexes were created for testing, debugging, and performance enhancement. Manual user acceptance testing was performed by randomly selecting five studies per data element (from a total of 109 data elements) from the AACT database. The values associated with each data element were tested for correctness and completeness by comparing them with the original source data from downloaded XML files. We also created integrated data dictionary tables as reference tables holding explicit data element definitions and system metadata (Tables S1 and S2).

During the course of database development, the NLM made several new data elements available for public download, some of which included information about the FDA (e.g., Section 801 clinical trials, studies with FDA-regulated interventions, and expanded-access studies). In addition to these, MeSH condition and intervention terms generated by the NLM algorithm were also made available for public download.

In XML files downloaded from ClinicalTrials.gov, the single data element *Study Design* contains a string of concatenated values for various different components of a study design, such as primary purpose, interventional model, observational model, allocation, endpoint classification, time perspective, and masking. While this format is well-suited for supporting information retrieval, it does not readily accommodate aggregate data analysis of the components within the *Study Design* data element. For this reason, data from *Study Design* was parsed into its components and stored in a separate table called DESIGNS. Additional data elements (*Design Name* and *Design Value*) were created to store all components of study design and their respective enumerated values. Values related to masking/blinding (e.g., *Single; Double-Blind*) were further parsed into their components, along with the list of corresponding masking subjects (*Participant*, *Investigator*, *Outcome Assessor*, and *Caregiver*).

Several challenges were encountered while loading the database, including foreign characters embedded in XML files with most of the data elements; these had to be replaced with character references (see Table 1 for examples).

**Table 1:** Escape characters and replacements.

Escape character	Replacement
'	'
"	"
&	&
"	>
<	<

Other circumstances that prompted several database design iterations included the facts that the maximum length for each data element noted by ClinicalTrials.gov's May 2010 Protocol Data Element Definitions document was not always consistent with the complete dataset, and one-to-one or one-to-many relationships between or among data elements were not obvious in the XML data type definition from ClinicalTrials.gov.

#### 1.3. Quality Assessment

**Figure 3:**
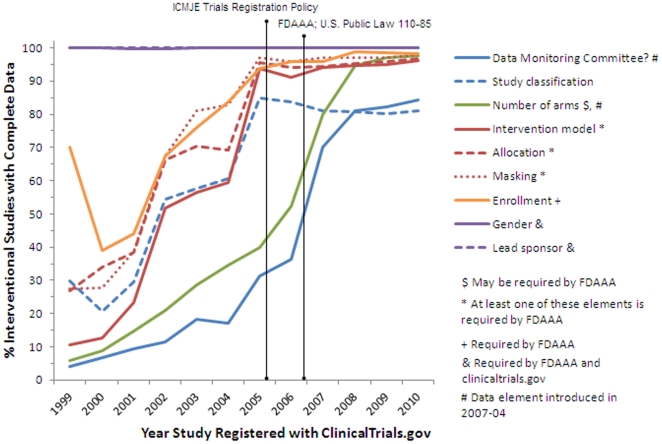
Percentage of interventional studies with complete data by registration year for selected data elements.

Of the 96,346 studies downloaded from ClinicalTrials.gov in September 2010, a total of 79,413 (82.4%) were interventional (i.e., a study in which an investigator following a protocol assigns research participants to receive specific interventions, as opposed to an observational study), 16,506 (17.1%) were observational, 107 (0.1%) were expanded-access, and 320 had no information about the study type. We analyzed selected data elements in interventional studies for completeness of data (e.g., a null value in the data element) and observed a trend toward increasing completeness of data over time. This trend appears to have been notably affected by two milestones in the history of ClinicalTrials.gov. In September 2004, the International Council of Medical Journal Editors (ICMJE) published a policy requiring registration of interventional trials as a condition of publication [3]. The ICMJE requirements took effect in September 2005, which may account for the increase in completeness for some data elements in 2005 (Figure 3).

In September 2007, the FDAAA [1] made the registration of interventional studies mandatory. This requirement took effect in December 2007 and may further account for increases in the completeness of data elements in the ClinicalTrials.gov dataset. In Figure 3, the data elements “data monitoring committee” and “number of arms” were not available at the time that earlier studies were registered. It is important to note that the presence of these data elements for studies pre-dating December 2007 reflect later updates performed by data providers.

#### 1.4. Changes in ClinicalTrials.gov's Protocol Data Element Definitions

The ClinicalTrials.gov Protocol Data Element Definitions (PDED) have evolved since the database was first launched. Although references containing individual protocol data element definitions are provided for submitters with each release of the definitions document, there is no document that tracks changes for all data elements for review as data specifications. These include changing enumerated values for a data element, revising a data element definition, making a particular data element publicly available, introducing a new data element, and entirely deleting a data element. However, more rigorous submission rules imposed by mandating organizations (e.g., NLM, FDA), such as those required by the FDAAA and ClinicalTrials.gov, appear to have had the greatest impact on the completeness of data.

Changes to a data element play a significant role in the analysis of study data. As we examined each data element's history, we noted that between September 2004 and July 2005 (a period spanning 3 releases of the PDED), and again in December 2007, the data element requirements were not documented in the definitions document. Other inconsistencies were also noted and later confirmed (Personal communication, Dr. Deborah Zarin and Mr. Nicholas Ide, February 18, 2011).

#### 1.5. A Public Resource

The AACT can be downloaded as Oracle extracts (.dmp file and text format output; available at https://www.trialstransformation.org/projects/improving-the-public-interface-for-use-of-aggregate-data-in-clinicaltrials.gov/aact-database-for-aggregate-analysis-of-clinicaltrials.gov). Additional documents are available to assist users in interpreting the data. The high-level data dictionary and a comprehensive data dictionary noted previously are included in the dataset file. The comprehensive data dictionary contains seven sections: 1) current variables, 2) enumerations, 3) constraints, 4) record counts, 5) database schema, 6) comprehensive change history, and 7) variable history dates. This document provides definitions, derivation of terms, data model structure and references, NLM and FDAAA requirements, and historical information for each data element in ClinicalTrials.gov to facilitate understanding of when variables were added, modified, or discontinued. The high-level data dictionary provides a summary view of the variables contained in the AACT database.

### 2. A Methodology to Regroup Studies in ClinicalTrials.Gov by Specialty

ClinicalTrials.gov contains studies from multiple clinical domains. While the AACT database facilitates the aggregate analysis of the entire dataset, it does not in itself support analysis within specific specialty domains. We therefore developed a methodology to re-group studies from ClinicalTrials.gov by clinical specialties as designated by the Department of Health and Human Services [14]. In doing so, we relied on MeSH condition terms and free-text disease condition terms associated with each study in the ClinicalTrials.gov database—a method that can be used to develop other specialized datasets for analysis.

#### 2.1. Use of MeSH Terminology in the ClinicalTrials.gov Database

**Figure 4:**
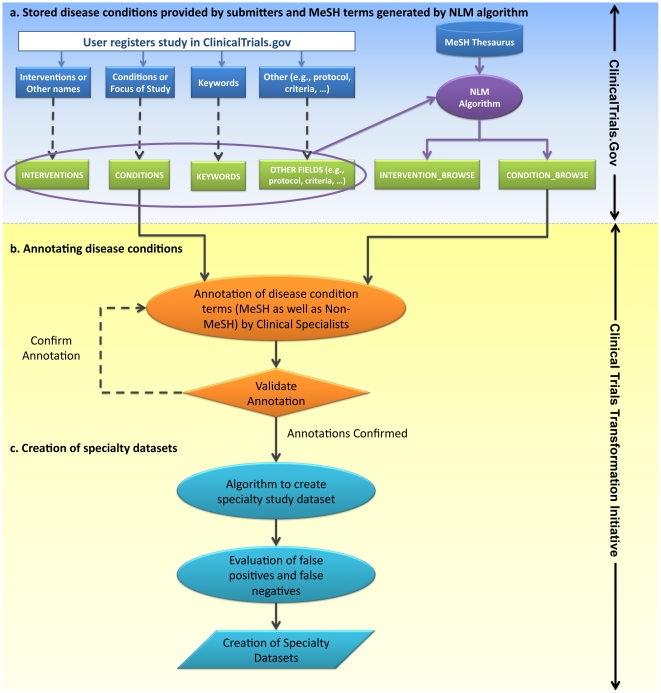
An overview of methodology and process of developing clinical specialty datasets. The INTERVENTIONS, CONDITIONS, and KEYWORDS tables consist of disease condition terms provided by data submitters that include both MeSH and non-MeSH terms. The INTERVENTION_BROWSE and CONDITION_BROWSE tables are populated by MeSH terms generated by NLM algorithm (a) Process illustrating how MeSH terms are created in ClinicalTrials.gov. Tables and data shown here does not represent entire ClinicalTrials.gov database (b) Process illustrating the annotation and validation of disease conditions (c) Process illustrating the creation of specialty datasets.

Data submitters (study sponsors or their designees) are requested to provide *Condition* and *Keywords* data as MeSH terms when registering a study. Additionally, an NLM algorithm also evaluates studies and applies MeSH terms according to the following steps: 1) study records are checked for the presence of a MeSH term, including synonyms and lexical variations; 2) weighted scores are computed for all matches, with exact matches, lexical variations, and synonyms receiving descending proportional weight; 3) very common terms are excluded to avoid confounding; 4) location by data element is considered and weighted in the term scoring process; and 5) terms with scores exceeding the cutoff value are applied to the respective studies. (Note that the output from steps 1 and 2 is used for both condition and intervention annotations; the field weights are different for each and divert terms into the target annotation type.) This method does not consider the natural-language context for matched terms or ontologically related concepts that would add specificity. Neither the terms from data submitters nor the NLM algorithm attempt to associate a term with a particular MeSH hierarchy. These resulting annotated MeSH terms are visible on the ClinicalTrials.gov website and populated in the *condition_browse* and *intervention_browse* fields in the downloaded XML file for each study. Figure 4 illustrates how MeSH terms are created in the ClinicalTrials.gov database.

#### 2.2. MeSH Disease Conditions Annotation

Condition and intervention terms in the MeSH thesaurus are arrayed in hierarchical branching structures, called *trees*; each branching point is referred to as a *node*. Nodes range from 1 (highest level) to 12 (lowest level) in the 2010 version of the MeSH thesaurus. For example, one high-level category that we used to classify studies by clinical specialty was *Diseases*. In the 2010 MeSH thesaurus, this category contains 23 subcategories (Table 2).

**Table 2:** MeSH Subject Headings, 2010—Diseases.

Bacterial Infections and Mycoses [C01]
Virus Diseases [C02]
Parasitic Diseases [C03]
Neoplasms [C04]
Musculoskeletal Diseases [C05]
Digestive System Diseases [C06]
Stomatognathic Diseases [C07]
Respiratory Tract Diseases [C08]
Otorhinolaryngologic Diseases [C09]
Nervous System Diseases [C10]
Eye Diseases [C11]
Male Urogenital Diseases [C12]
Female Urogenital Diseases and Pregnancy Complications [C13]
Cardiovascular Diseases [C14]
Hemic and Lymphatic Diseases [C15]
Congenital, Hereditary, and Neonatal Diseases and Abnormalities [C16]
Skin and Connective Tissue Diseases [C17]
Nutritional and Metabolic Diseases [C18]
Endocrine System Diseases [C19]
Immune System Diseases [C20]
Disorders of Environmental Origin [C21]
Animal Diseases [C22]
Pathological Conditions, Signs and Symptoms [C23]
Available at: http://www.nlm.nih.gov/mesh/trees.html

In order to create specialty datasets from the larger AACT dataset, we selected four high-level MeSH nodes from the thesaurus to serve as an initial basis for identifying studies by clinical specialty. Reviewers with relevant subject matter expertise annotated MeSH terms from the following high-level nodes: 1) *Diseases*; 2) *Analytical*, *Diagnostic and Therapeutic Techniques and Equipment*; 3) *Psychiatry and Psychology*; and 4) *Phenomena and Processes*.

A total of 18,491MeSH IDs associated with 9031 MeSH terms were reviewed and annotated by clinical specialists belonging to one of the 13 clinical specialties and five sub-specialties, which were selected on the basis of availability of faculty representation and volunteers at Duke, as well as intention to analyze subsets of data by clinical specialty. Participating specialty annotations included cardiology, dermatology, endocrinology, gastroenterology, immunology/ rheumatology, infectious diseases, mental health, nephrology, neurology, oncology, otolaryngology, pulmonary medicine, reproductive medicine, while subspecialty annotations included peripheral vascular disease, peripheral arterial disease, diabetes, thyroid disease, and bone disease. The association of terms with clinical specialties was performed in the context of the anticipated analysis of the data subset for respective specialties. The results of this extension to the AACT database, including specialty tags, will be shared in future publications.

#### 2.3. Validation of Inconsistently Annotated MeSH Terms and Limitations of Using the MeSH Hierarchy

A term occurring at a particular node “node *x*” (parent) may have several branches (children) at node *x+1* that provide a finer classification of the node-*x* term. Clinical specialists were advised to review the hierarchy of an individual MeSH term during the annotation process. Annotated MeSH descriptors were programmatically reviewed for hierarchical inconsistencies in order to maintain the logical relationship between parent and child MeSH descriptors. Tag validity was evaluated by a process based on annotation rules. In general, selection or negation of a parent MeSH term should match with *all* subsequent child MeSH terms below that node. Hierarchical inconsistencies in MeSH annotations were flagged and accepted after further review and confirmation by clinical specialists. The anticipated inconsistency of the MeSH hierarchical structure with clinical specialty groupings was confirmed in the validation process. Table 3 shows the frequency of parent terms that did not match with annotations for their children terms.

**Table 3:** Frequency of intermediate terms and top node terms that did not match annotations of lower-level terms.

Specialty	n/N (%)
Cardiology	172/5264 (3.3%)
Oncology	284/5264 (5.4%)
Mental health	93/5264 (1.8%)

n = number of intermediate- and top-node MeSH terms for a given specialty that do not match the annotations of their lower-level terms. N = total number of intermediate- and top-node MeSH terms.

**Figure 5:**
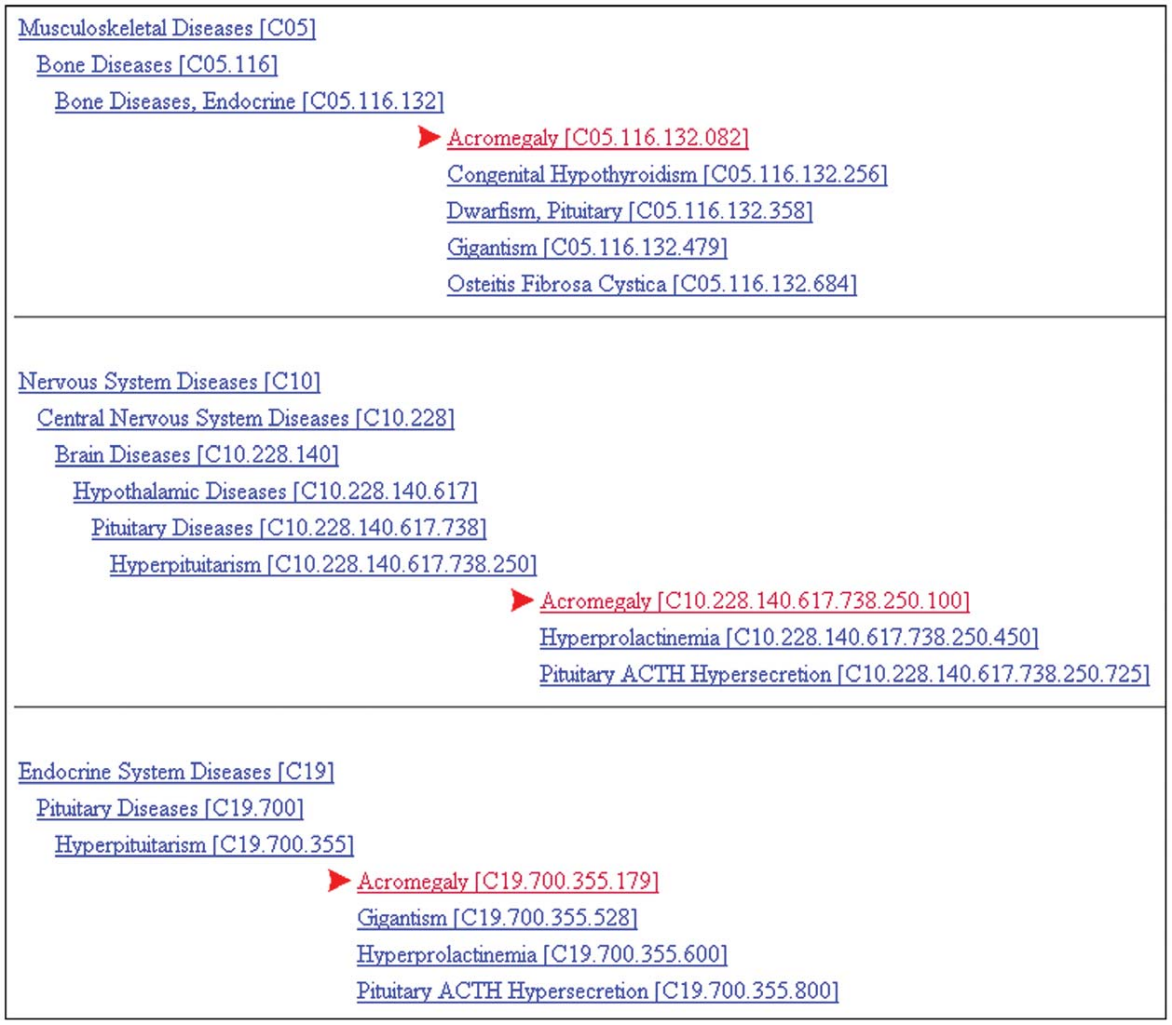
MeSH trees for acromegaly. Source: 2010 online MeSH thesaurus (available: http://www.nlm.nih.gov/cgi/mesh/2010/MB_cgi).

Further, a term might appear within more than one tree. For example, the MeSH term *Acromegaly* appears as part of multiple trees within the topmost MeSH hierarchical category of *Diseases* (Figure 5).

Depending on its hierarchical location, its context could fall under *Musculoskeletal Diseases*, *Nervous System Diseases*, or *Endocrine System Diseases*. Unfortunately, there currently is no way to differentiate among different tree numbers (MeSH IDs) for the same MeSH term. If a study contained the term *Acromegaly*, the three associated MeSH IDs could have conflicting tags (e.g., *No*, *No*, *Yes*) for a given specialty. This might result in erroneously including this study in a particular specialty dataset. As an additional validation check, all MeSH terms that had conflicting tags, as in the example above, were flagged and allowed to be adjudicated by clinical specialists.

Tagging was summarized by MeSH term. For a given MeSH term, if all MeSH IDs had a Y tag (“yes” or “true”), then the MeSH term was given a Y; if all MeSH IDs had an N tag (“no” or “false”), then the MeSH term was given a N tag; and if there was a mix of Y and N tags the term was given an A tag (“ambiguous”).

#### 2.4. Free-text Disease Conditions (non-MeSH condition terms): Annotation and Validation

In order to ascertain the condition being investigated in a given study, we also used the free-text condition terms provided by data submitters. These terms are visible on the ClinicalTrials.gov website and populated in the Condition field in the downloaded XML file for each study. Non-MeSH condition terms that appeared in five or more studies were also selected for specialty classification from interventional studies registered after September 27, 2007 (n = 40,970). These terms were reviewed by two independent clinicians from each relevant specialty; disagreements were adjudicated by a third independent reviewer.

We elected to use both MeSH and non-MeSH disease condition terms for the following reasons: first, over 10% of studies do not have *condition_browse* mesh_terms; second, common terms may be excluded from the *condition_browse* mesh_terms annotation; and third, because of the potential for duplication or mismatch described above, reliance on indexing by MeSH term alone does not suffice for re-grouping studies in ClinicalTrials.gov by clinical specialty.

#### 2.5. Algorithm for Classifying Clinical Discipline

We used a combination of rules representing disease conditions and MeSH terms for classifying clinical specialty within interventional studies. We only included trials registered with ClinicalTrials.gov after September 27, 2007. The final list of annotated disease condition terms (MeSH and free-text) was used as a lookup table to create study datasets for individual specialties.

**Figure 6:**
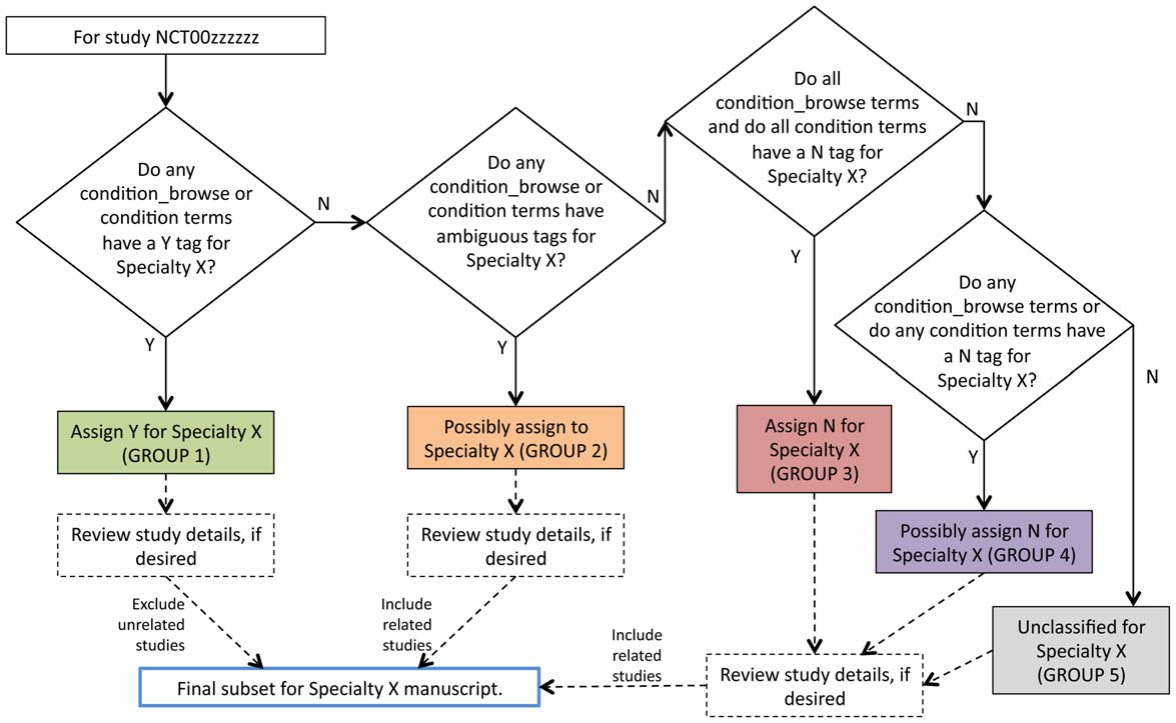
Rules for deciding whether a given study belongs to a given specialty.

For each specialty, studies were grouped according to the following rules (Figure 6):

Group 1: Include a study in this group if any of its MeSH terms from the CONDITION_BROWSE table or condition terms were annotated with a Y (“yes” or “true”) for the specialty.

Group 2: Include a study in this group if a) it is not in Group 1, and b) any of its MeSH terms from the CONDITION_BROWSE table or condition terms were annotated with an A (“ambiguous”) for the specialty.

Group 3: Include a study in this group if a) it is not in Groups 1 or 2, and b) all of its MeSH terms from the CONDITION_BROWSE table and all of its condition terms were annotated with an N (“no” or “false”) for the specialty.

Group 4: Include a study in this group if a) it is not in Groups 1, 2. or 3, and b) any of its MeSH terms from the CONDITION_BROWSE table or any of its condition terms were annotated with an N (“no” or “false”) for the specialty.

Group 5: Include all studies in this group that are not in Groups 1, 2, 3, or 4.

For the validation results reported in this manuscript, the cardiology, oncology, and mental health groups correspond to Group 1. A single study could be classified as belonging to multiple clinical disciplines. For the validation results reported below, studies in Groups 3 and 4 were pooled and correspond to studies not classified as not-cardiology, not-oncology, and not-mental health, respectively. For purposes of investigations focused on each respective clinical specialty, Groups 1 and 2 were pooled. Based on custom inclusion/exclusion criteria for these investigations, some of these studies were excluded from the final specialty datasets.

A different method was used to identify relevant studies for the specialties of genomics and pediatrics. For the genomics dataset, observational studies were also included, and condition fields, intervention fields, and other relevant fields were searched for terms such as “gene,” “genomic,” and “DNA.” Pediatric studies were defined as those that restricted enrollment to a pediatric population (i.e., maximum age of 18 years). The identification of the sets of genomics and pediatrics studies has not been validated by the methods described in the following section.

### 3. Quality Assessment of Specialty Classification System Using Cardiology, Mental Health, and Oncology Datasets

We evaluated the accuracy of the classification algorithm described above by comparing it with classifications provided by expert clinical review of classifications for cardiology, oncology, and mental health trials. These three specialties were chosen because together they represent approximately 39% (15,907) of all interventional trials (*n* = 40,970) registered in the ClinicalTrials.gov registry from September 27, 2007 to September 27, 2010. The expert review was performed to ensure that the allocation of studies into clinical domains would be valid for the intended analysis purpose of quantifying the distribution of studies by clinical discipline and describing and comparing the characteristics of studies across specialties.

#### 3.1. Manual Study Classification

To estimate misclassification rates for disease specialty classifications, a random sample of 1000 interventional studies registered with ClinicalTrials.gov within the relevant time period was selected from the clinical specialty datasets. The brief title, brief description, keywords, and study conditions were provided to a group of seven clinical specialists, who were also given a link to the full record in ClinicalTrials.gov if they needed more information to classify the study. The 1000-study sample was divided into batches of 200 studies. Each batch was reviewed by two clinical specialists (designated “A” or “B” for that particular batch; see Table 4) who indicated “yes,” “no,” or “unknown” for each study within the cardiology, oncology, and mental health classifications, respectively. Disagreements between reviewers were adjudicated by an independent eighth reviewer. Adjudicated results were compared with the specialty datasets to determine misclassification rates.

**Table 4:** Number of studies reviewed by each set of clinician reviewers.

Reviewer A ID	Reviewer B ID	Studies reviewed (n)
Clinician 1	Clinician 2	200
Clinician 1	Clinician 3	400*
Clinician 4	Clinician 5	200
Clinician 6	Clinician 7	200

* The combination of Clinician 1 (“A”) and Clinician 3 (“B”) together reviewed 2 batches of studies.

#### 3.2. Estimation of Misclassification Errors

We used the algorithm described in the previous section to determine the predicted clinical domain for each study in the random sample (e.g., “yes” or “no” for cardiology), which was compared against the classification provided by the adjudicated expert review (Table 5). The studies in each cell of Table 5 were counted and the results for each clinical domain were considered independently. Two types of misclassification errors were considered. A false positive error is made if the algorithm classifies a study as *Y* (“Yes”) but in truth the study is an *N* (“No”). The “C” cell in indicates the false positive errors. A false negative error is made if the algorithm classifies a study as *N* but in truth the study is a *Y*. The “B” cell indicates the false negative errors.

**Table 5:** Contingency table for identifying misclassification errors.

	Algorithm					
		Yes (Y)	No (N)	Ambiguous	Unclassified	Total
**Manual review**	Yes (Y)	A	**B**	G	H	A+B+G+H
	No (N)	**C**	D	I	J	C+D+I+J
	Unknown	E	F	K	L	E+F+K+L
Total		A+C+E	B+D+F	G+I+K	H+J+L	T

The overall misclassification error rate divides the total number of errors by the total number of studies reviewed. The false positive rate was determined using two methods: in the first, the false-positive rate was calculated among studies classified as *N* by manual review; in the second, the false-positive rate was calculated among studies classified as *Y* by the algorithm. The false-negative rate was evaluated in similar fashion: by dividing the number of false negatives by the number of studies classified as *Y* by manual review, or by the number of studies classified as N by the algorithm.

#### 3.3. Sampling Approach and Required Sample Size

Several factors informed our decision to select a sample size of approximately 1000 studies. First, if the true rate of misclassification was relatively low, we would need a correspondingly large number of studies to detect misclassifications. Second, because the precision of the estimate of the error rate increases with the sample size, we selected a sample size likely to provide reasonable precision. Third and finally, in order to ensure sufficient representation from each clinical domain, we needed to sample a large number of studies from the overall database (e.g., cardiology was expected to comprise <10% of the total database).

#### 3.4. Comparisons of Algorithmic Classification with Manual Classification

Classification of sampled studies by manual review as cardiology, oncology, or mental health was compared with classification by algorithm. Table 6 shows the comparisons for manual vs. algorithmic classification for cardiology, oncology, and mental health. The results of comparisons are summarized in Table 7. Rates of disagreement between clinical reviewers are presented in Table 8.

**Table 6:** Classification of studies: algorithmically vs. manually.

CARDIOLOGY						
	Algorithm					
		N	Y	Ambiguous	Unclassified	Total
	N	836	18	1	49	904
**Manual review**	Y	21	72	0	2	95
	Unknown	1	0	0	0	1
	Total	858	90	1	51	1,000

ONCOLOGY						
	Algorithm					
		N	Y	Ambiguous	Unclassified	Total
	N	700	4	1	49	754
**Manual review**	Y	7	237	0	2	246
	Unknown	0	0	0	0	0
	Total	707	241	1	51	1,000

MENTAL HEALTH						
	Algorithm					
		N	Y	Ambiguous	Unclassified	Total
	N	838	21	8	51	918
**Manual review**	Y	10	72	0	0	82
	Unknown	0	0	0	0	0
	Total	848	93	8	51	1,000

**Table 7:** Comparison between manual classification and algorithmic classification for cardiology, oncology, and mental health.

	Cardiology	Oncology	Mental Health
% Specialty by manual review	9.5%	24.6%	8.2%
% Specialty by algorithm*	9.5%	25.4%	9.9%
False positives†			
Among studies classified as N by manual review	2.0%	0.5%	2.3%
Among studies classified as Y by algorithm	20.0%	1.7%	22.6%
False negatives‡			
Among studies classified as Y by manual review	22.1%	2.8%	12.2%
Among studies classified as N by algorithm	2.4%	1.0%	1.2%
Overall incorrectly classified studies	4.2%	1.2%	3.3%
Overall ambiguous studies	0.1%	0.1%	0.8%
Overall unclassified studies	5.1%	5.1%	5.1%

* Excluding unclassified & ambiguous from denominator.

† Studies that were incorrectly included in a given specialty (e.g. non-cardiology studies classified as cardiology).

‡ Studies that were incorrectly excluded from a given specialty (e.g. cardiology studies classified as non-cardiology).

**Table 8:** Summary of disagreements between clinical specialty reviewers in study classification.

	Disagreement* n/N (%)		
Reviewers A&B	Cardiology	Oncology	Mental health
Reviewers 1 & 2	12/200 (6.0%)	9/200 (4.5%)	16/200 (8.0%)
Reviewers 1 & 3	20/400 (5.0%)	6/400 (1.5%)	18/400 (4.5%)
Reviewers 4 & 5	18/200 (9.0%)	11/200 (5.5%)	14/200 (7.0%)
Reviewers 6 & 7	18/200 (9.0%)	9/200 (4.5%)	18/200 (9.0%)
Overall	68/1,000 (6.8%)	35/1,000 (3.5%)	66/1,000 (6.6%)

* Defined as any difference in classification of a study by the two reviewers of that study.

#### 3.5. Evaluating Use of NLM-generated MeSH Terms in Addition to Disease Condition Terms for Specialty Classification

As described above, specialty classification uses data from two fields: the *condition* field, which contains the disease conditions provided by the data submitter and accommodates a combination of MeSH and non-MeSH terms; and the *condition_browse* field, which contains only MeSH terms generated by the NLM algorithm. To evaluate the reliability of using *condition_browse* for classifying studies into specialty groups, we compared study datasets created using only NLM-generated MeSH disease condition terms with those created using only submitted conditions. We used cardiology, oncology, and mental health annotations to perform comparisons between methods and found that the differences were within 4.9% for these specialty datasets (cardiology within 1.1%; oncology within 2.5%; mental health 4.9%). Table 9 shows a comparative analysis using two sources (*condition* and *condition_browse*).

**Table 9:** Summary of results of comparison between *condition_browse* and *condition* data by specialty classification.

	Cardiovascular		Oncology		Mental health	
	Condition browse	Condition	Condition browse	Condition	Condition browse	Condition
% Specialty by manual review	9.5%	9.5%	24.6%	24.6%	8.2%	8.2%
% Specialty by algorithm*	8.6%	9.1%	27.3%	24.8%	8.3%	9.1%
False positives†						
Among studies classified as N by manual review	1.4%	1.5%	0.4%	0.3%	1.2%	1.5%
Among studies classified as Y by algorithm	17.8%	18.2%	1.3%	0.9%	15.9%	18%
False negatives‡						
Among studies classified as Y by manual review	23.2%	22.1%	2.8%	4.5%	8.5%	13.4%
Among studies classified as N by algorithm	2.8%	2.7%	1.7%	1.7%	1.4%	1.4%
Overall incorrectly classified studies	4.1%	4.2%	1.2%	1.5%	2.2%	3.0%
Overall ambiguous studies	0.0%	0.1%	0.3%	0.0%	1.5%	0.8%
Overall unclassified studies	15.5%	14.9%	15.5%	14.9%	15.5%	14.9%

* excluding unclassified & ambiguous from denominator.

† Studies that were incorrectly included in a given specialty (e.g., non-cardiology studies classified as cardiology).

‡ Studies that were incorrectly excluded from a given specialty (e.g., cardiology studies classified as non-cardiology).

## Results and Discussion

To our knowledge, the present study represents the first report of an attempt to create and maintain a version of ClinicalTrials.gov data for aggregate analysis and public use of the data—undertakings that constitute a significant enhancement of the ClinicalTrials.gov database. Specifically, we have described the procurement and generation of an integrated database; the parsing of the database's *Study Design* variable into a format that can be used for analysis; the creation of a change history in the underlying data element definitions; and the display of basic trends in data quality metrics.

Our novel system for classifying studies by clinical specialty is a result of combining technology with medical subject matter expertise, based on extensive data curation that incorporates coding by physicians representing relevant clinical specialties, as well as use of MeSH taxonomy and submitted disease conditions. Applying this methodology to complete a taxonomy by clinical specialty would facilitate policy-oriented investigations, such as 1) future research policy studies aimed at evaluating disparities in the geographical distribution of clinical trials and of biomedical research literature in relation to the local burden of diseases [15]; 2) the comparative evolution in the number of trials and resulting publications over time by clinical specialties [16]; 3) the progression of interdisciplinarity in medical specialties (by examining the degree of overlap in MeSH terms over time); and 4) the comparative distribution of evidence levels across specialties, focusing on areas historically characterized by low levels of evidence. Each of these topics is crucial, not only for ensuring alignment between regional and global healthcare needs, but also for defining evidence-based strategic plans in relation to national and regional biomedical research policies.

Despite the significant value provided by the AACT, this resource has limitations. First, ClinicalTrials.gov was primarily intended to serve as a public data repository and was not designed to support aggregate analysis. Second, the original MeSH classification was not created to accommodate hierarchical arrangement of clinical specialties and a given condition may be stored in multiple locations. Because the current MeSH structure does not allow differentiation among clinical specialties, false positive results may occur when attempting to query the database for a given condition. Third, because the methodology developed to annotate the AACT database by clinical specialty relied on a group of experts exclusively from Duke University, further validation would be appropriate. Fourth, the process for curation and development of the taxonomy were both time- and resource-intensive. For this reason, further investigation should explore methods for streamlining this process, potentially distributed across a large, open curation community. Fifth, the use of standard ontologies and vocabularies from all clinical disciplines would be ideal for encompassing a complete specialty classification. Sixth and finally, while we believe that our study classification methodologies have the potential to facilitate policy-oriented investigations, the usefulness of our approach in these arenas will need to be confirmed by more formal validation processes.

Future development for this database could expand into a number of areas. First, the curated database and, when completed, the accompanying clinical specialty taxonomy, could be represented using the Resource Description Framework (RDF) [17] to enable merging with the linkedCT [9] version of the ClinicalTrials.gov dataset and other RDF resources. Second, possible additions to the database include the standardization of sponsor lists, interventions, outcome measures, universities, and the connection to geolocation datasets such as GeoNames [18]. Importantly, future additions should be driven by the value they might provide to research policy makers [19], ultimately improving the quality of biomedical research and, consequently, the healthcare delivered to patients.

## Supporting Information

Table S1 ClinicalTrials.gov High-level Data Dictionary.
(PDF)

Table S2 ClinicalTrials.gov Comprehensive Data Dictionary.
(XLSX)
