# Supplementary material for: The Database for Aggregate Analysis of ClinicalTrials.gov (AACT) and Subsequent Regrouping by Clinical Specialty
Source: PLoS One. 2012 Mar 16;7(3):e33677. doi: 10.1371/journal.pone.0033677 (PMC3306288; doi:10.1371/journal.pone.0033677)
Supplement: Table S1 — ClinicalTrials.gov High-level Data Dictionary. (PDF) [file pone.0033677.s001.pdf]

Data Dictionary (V2.0)

Comments on 'CTTI_Notes' column: CTTI notes are provided for those variables that are either derived from ClinicalTrials.gov data elements (e.g. 'Design_Name' and 'Design_Value' are derived from 'Study_Design'), or are not available in ClinicalTrials.gov Protocol Data Element Definitions (e.g. , 'Agency_Class', 'First_Received_Results_Date', and 'MeSH_Term' etc.), or are defined primarily for CTTI's database purposes (e.g. system generated sequential IDs).

Data Element Requirements
'NLM Required' column: 'Yes' if ClinicalTrials.gov requires a user to enter a value.
'FDAAA Required' column: 'Yes' if user is required to enter a value in order to comply with US Public Law 110-85, Section 801. 'Maybe' if User may be required to enter a value in order to comply with US Public Law 110-85, Section 801.
Within 'NLM Definitions' Column:
 * --- Required by ClinicalTrials.gov
 FDAAA --- Required to comply with US Public Law 110-85, Section 801
 (FDAAA) --- May be required to comply with US Public Law 110-85, Section 801

Variable Name	Variable Label	Value List	Entity Name	NLM Required	FDAAA Required	NLM Definitions	CTTI Definitions	Variable Source
address_id	Primary Key for ADDRESSES		ADDRESSES	N/A	N/A		Numeric sequential ID generated by system. ADDRESS_ID_SEQ. A record was generated in ADDRESSES table for every NCT_ID (unique study ID), even when there is no data under address (<city>, <state>, <zip>, <country>) for that NCT_ID from ClinicalTrials.gov dataset. In this case the record has NULL values for city, state, zip, and country variables.	CTTI
city	City		ADDRESSES	Yes	Maybe	City		NLM
country	Country		ADDRESSES	Yes	Maybe	Country		NLM
facility_id	Foreign Key for FACILITIES		ADDRESSES	N/A	N/A		Numeric sequential ID generated by system. FACILITY_ID_SEQ	CTTI
nct_id	ClinicalTrials.gov registry number		ADDRESSES	No	No	N/A	The NCT number is a unique number assigned by clinicaltrials.gov to each clinical trial.	NLM
state	State		ADDRESSES	Yes	Maybe	State		NLM
zip	Zip		ADDRESSES	No	No	Zip		NLM
arm_group_id	Primary Key for ARM_GROUPS		ARM_GROUPS	N/A	N/A		Numeric sequential ID generated by system. ARM_GROUP_ID_SEQ	CTTI

Variable Name	Variable Label	Value List	Entity Name	NLM Required	FDAAA Required	NLM Definitions	CTTI Definitions	Variable Source
arm_group_label	Arm or Group Label		ARM_GROUPS	Yes	Maybe	Arm Label - the short name used to identify the arm. (Limit: 62 characters).<br>* (FDAAA)<br><br>Group/Cohort Label - the short name used to identify the group. (Limit: 62 characters)<br>*		NLM
arm_group_type	Arm Type	Experimental<br>Sham Comparator<br>Other<br>Control<br>Exposure Comparison<br>Treatment Comparison<br>Case<br>No intervention<br>Placebo Comparator<br>Active Comparator	ARM_GROUPS	Yes	Maybe	Arm Type (Experimental, Active Comparator, Placebo Comparator, Sham Comparator, No intervention, Other)	Although the attribute's name is arm_group_type, the data is available only for "Arm Type".	NLM
description	Arm or Group Description		ARM_GROUPS	No	Maybe	Arm Description (FDAAA): Brief description of the arm. This element may not be necessary if the associated intervention descriptions contain sufficient information to describe the arm. (Limit: 999 characters)<br><br>Group/Cohort Description: Explanation of the nature of the study group (e.g., those with a condition and those without a condition; those with an exposure and those without an exposure). Note that the overall study population should be described under Eligibility. (Limit: 1000 characters)		NLM
nct_id	ClinicalTrials.gov registry number		ARM_GROUPS	No	No	N/A	The NCT number is a unique number assigned by clinicaltrials.gov to each clinical trial.	NLM

Variable Name	Variable Label	Value List	Entity Name	NLM Required	FDAAA Required	NLM Definitions	CTTI Definitions	Variable Source
authority	Oversight Authorities		AUTHORITIES	Yes	No	The name of each national or international health organization with authority over the protocol. Use the following format for each authority: country: organization name.<br><br>Examples:<br>United States: Institutional Review Board<br>United States: Food and Drug Administration<br>Germany: Federal Institute for Drugs and Medical Devices<br>Australia: Therapeutic Goods Administration		NLM
authority_id	Primary Key for AUTHORITIES		AUTHORITIES	N/A	N/A		Numeric sequential ID generated by system. AUTHORITY_ID_SEQ	CTTI
nct_id	ClinicalTrials.gov registry number		AUTHORITIES	No	No	N/A	The NCT number is a unique number assigned by clinicaltrials.gov to each clinical trial.	NLM
acronym	Acronym		CLINICAL_STUDY	No	No	Acronym or initials used to identify this study, if applicable. Enter only the acronym. If supplied, the acronym is automatically displayed in parentheses following the brief title. (Limit: 14 characters)		NLM
biospec_descr	Biospecimen Description		CLINICAL_STUDY	No	No	Specify all types of biospecimens to be retained (e.g., whole blood, serum, white cells, urine, tissue). (Limit: 1000 characters)		NLM
biospec_retention	Biospecimen Retention	None Retained<br>Samples With DNA<br>Samples Without DNA	CLINICAL_STUDY	No	No	Retention status		NLM
brief_summary	Brief Summary		CLINICAL_STUDY	Yes	Yes	Short description of the protocol intended for the lay public. Include a brief statement of the study hypothesis. (Limit: 5000 characters)		NLM
brief_title	Brief Title		CLINICAL_STUDY	Yes	Yes	Protocol title intended for the lay public. (Limit: 300 characters)		NLM
completion_date	Study Completion Date		CLINICAL_STUDY	No	No	Final date on which data was (or is expected to be) collected. Use the Type menu (Anticipated/Actual).		NLM

Variable Name	Variable Label	Value List	Entity Name	NLM Required	FDAAA Required	NLM Definitions	CTTI Definitions	Variable Source
completion_date_type	Study Completion Date Type	Actual Anticipated	CLINICAL_STUDY	No	No	A "Type" menu is also included, with options Anticipated and Actual. For active studies, set Type to Anticipated and specify the expected completion date, updating the date as needed over the course of the study. Upon study completion, change Type to Actual and update the date if necessary.		NLM
criteria	Eligibility Criteria		CLINICAL_STUDY	Yes	Yes	Summary criteria for participant selection. The preferred format includes lists of inclusion and exclusion criteria as shown below. (Limit: 15,000 characters)		NLM
detailed_description	Detailed Description		CLINICAL_STUDY	No	No	Extended description of the protocol, including more technical information (as compared to the Brief Summary) if desired. Do not include the entire protocol; do not duplicate information recorded in other data elements, such as eligibility criteria or outcome measures. (Limit: 32,000 characters)		NLM
download_date	Download		CLINICAL_STUDY	No	No	N/A	Date of download from NLM	NLM
download_date_dt	Download Date (Parsed)		CLINICAL_STUDY	No	No		Date of download from NLM, free text date converted to DATE type	CTTI
enrollment	Enrollment		CLINICAL_STUDY	Yes	Yes	Target or Actual Number of Subjects. Number of subjects in the trial.		NLM
enrollment_type	Enrollment Type	Actual Anticipated	CLINICAL_STUDY	Yes	Yes	A "Type" menu is also included, with options Anticipated and Actual. For active studies, set Type to Anticipated and specify the expected enrollment, updating the number as needed over the course of the study. Upon study completion, change Type to Actual and update the enrollment if necessary.		NLM
firstreceived_date	First Received Date		CLINICAL_STUDY	No	No	N/A	Date of data first available to public (approximate)	NLM
firstreceived_results_date	First Received Results Date		CLINICAL_STUDY	No	No	N/A	This tag is available only of those studies that have results and indicates the date when the results were first received.	NLM

Variable Name	Variable Label	Value List	Entity Name	NLM Required	FDAAA Required	NLM Definitions	CTTI Definitions	Variable Source
gender	Gender	Both<br>Female<br>Male	CLINICAL_STUDY	Yes	Yes	Physical gender of individuals who may participate in the protocol.		NLM
has_dmc	Data Monitoring Committee?	yes<br>no	CLINICAL_STUDY	No	No	Indicate whether a data monitoring committee has been appointed for this study. The data monitoring committee (board) is a group of independent scientists who are appointed to monitor the safety and scientific integrity of a human research intervention, and to make recommendations to the sponsor regarding the stopping of the trial for efficacy, for harms or for futility. The composition of the committee is dependent upon the scientific skills and knowledge required for monitoring the particular study.		NLM
has_expanded_access	Has Expanded Access?	yes<br>no	CLINICAL_STUDY	No	Yes	Indicate whether any non-protocol access is to be provided for the investigational drug or device. If so, an Expanded Access record should also be created for this IND/IDE.		NLM
healthy_volunteers	Accepts Healthy Volunteers?		CLINICAL_STUDY	No	Yes	Indicate if persons who have not had the condition(s) being studied or otherwise related conditions or symptoms, as specified in the eligibility requirements, may participate in the study.		NLM
is_fda_regulated	FDA Regulated Intervention?	yes<br>no	CLINICAL_STUDY	No	Maybe	Indicate whether this trial includes an intervention subject to US Food and Drug Administration regulation under section 351 of the Public Health Service Act or any of the following sections of the Federal Food, Drug and Cosmetic Act: 505, 510(k), 515, 520(m), and 522.		NLM

Variable Name	Variable Label	Value List	Entity Name	NLM Required	FDAAA Required	NLM Definitions	CTTI Definitions	Variable Source
is_section_801	Is section 801?	yes<br>no	CLINICAL_STUDY	No	Maybe	If this trial includes an FDA regulated intervention, indicate whether this is an "applicable clinical trial" as defined in US Public Law 110-85, Title VIII, Section 801. Briefly, applicable drug trials include controlled clinical investigations, other than Phase I investigations, of a drug or biologic subject to US FDA regulation. Applicable device clinical trials are controlled trials with health outcomes of devices subject to FDA regulation, other than small feasibility studies, and pediatric postmarket surveillance.		NLM
lastchanged_date	Last Changed Date		CLINICAL_STUDY	No	No	N/A	Date of last entry change.	NLM
maximum_age	Maximum Age		CLINICAL_STUDY	Yes	Yes	Maximum age of participants. Provide a number and a unit of time (years, months, weeks, days, hours or minutes). Select "N/A (No limit)" if no maximum age is indicated.		NLM
minimum_age	Minimum Age		CLINICAL_STUDY	Yes	Yes	Minimum age of participants. Provide a number and select a unit of time (years, months, weeks, days, hours or minutes). Select "N/A (No limit)" if no minimum age is indicated.		NLM
nct_alias	Alternate NCT_ID		CLINICAL_STUDY	No	No	N/A	The NCT_alias contains alternate NCT_ID numbers. Studies were formerly associated with these IDs at one time.	NLM
nct_id	ClinicalTrials.gov registry number		CLINICAL_STUDY	No	No	N/A	The NCT number is a unique number assigned by clinicaltrials.gov to each clinical trial.	NLM
number_of_arms	Number of Arms		CLINICAL_STUDY	No	Maybe	Number of intervention groups (enter 1 for single-arm study).		NLM
number_of_groups	Number of Groups		CLINICAL_STUDY	Yes	No	Number of study groups/cohorts. Enter 1 for a single-group study. Many observational studies have one group/cohort; case control studies typically have two.		NLM

Variable Name	Variable Label	Value List	Entity Name	NLM Required	FDAAA Required	NLM Definitions	CTTI Definitions	Variable Source
official_title	Official Title		CLINICAL_STUDY	No	No	Official name of the protocol provided by the study principal investigator or sponsor. (Limit: 600 characters)		NLM
org_study_id	Organization's Unique Protocol id		CLINICAL_STUDY	Yes	Yes	Unique identification assigned to the protocol by the sponsoring organization, usually an accession number or a variation of a grant number. Multiple studies conducted under the same grant must each have a unique number. (Limit: 30 characters)		NLM
overall_status	Overall Recruitment Status	Not yet recruiting<br>Active, not recruiting<br>Suspended<br>Withdrawn<br>No Longer Available<br>Approved for marketing<br>Temporarily not available<br>Available<br>Terminated<br>Completed<br>Enrolling by invitation<br>Recruiting	CLINICAL_STUDY	Yes	Yes	Overall accrual activity for the protocol.		NLM
phase	Study Phase	N/A<br>Phase 4<br>Phase 3<br>Phase 2/Phase 3<br>Phase 2<br>Phase 1/Phase 2<br>Phase 1<br>Phase 0	CLINICAL_STUDY	Yes	Yes	Phase of investigation, as defined by the US FDA for trials involving investigational new drugs		NLM

Variable Name	Variable Label	Value List	Entity Name	NLM Required	FDAAA Required	NLM Definitions	CTTI Definitions	Variable Source
primary_completion_date	Primary Completion Date		CLINICAL_STUDY	No	Yes	As specified in US Public Law 110-85, Title VIII, Section 801, with respect to an applicable clinical trial, the date that the final subject was examined or received an intervention for the purposes of final collection of data for the primary outcome, whether the clinical trial concluded according to the prespecified protocol or was terminated. A "Type" menu is also included, with options Anticipated and Actual. For active studies, set Type to Anticipated and specify the expected completion date, updating the date as needed over the course of the study. Upon study completion, change Type to Actual and update the date if necessary.		NLM
primary_completion_date_type	Primary Completion Date Type	Actual<br>Anticipated	CLINICAL_STUDY	No	Yes	A "Type" menu is also included, with options Anticipated and Actual. For active studies, set Type to Anticipated and specify the expected completion date, updating the date as needed over the course of the study. Upon study completion, change Type to Actual and update the date if necessary.		NLM
sampling_method	Sampling Method	Probability Sample<br>Non-Probability Sample	CLINICAL_STUDY	Yes	No	For observational studies only, select one and explain in Detailed Description.		NLM
source	Source		CLINICAL_STUDY	No	No	N/A	Similar to Lead sponsor, but may indicate who the data was submitted on behalf of, or a subsidiary of the sponsor.	NLM
start_date	Study Start Date		CLINICAL_STUDY	No	Yes	Date that enrollment to the protocol begins.		NLM

Variable Name	Variable Label	Value List	Entity Name	NLM Required	FDAAA Required	NLM Definitions	CTTI Definitions	Variable Source
study_design	Study Design		CLINICAL_STUDY	Yes (Interventional Study Design)<br><br>No (Primary Purpose)<br><br>No (Intervention Model)<br><br>No (Allocation)<br><br>No (Study Classification)<br><br>Yes (Observational Study Model)<br><br>Yes (Time Perspective)	Maybe (Interventional Study Design)<br><br>Yes (Primary Purpose)<br><br>Maybe (Intervention Model)<br><br>Maybe (Allocation)<br><br>No (Study Classification)<br><br>No (Observational Study Model)<br><br>No (Time Perspective)	Interventional or Observational study design		NLM
study_pop	Study Population Description		CLINICAL_STUDY	Yes	No	For observational studies only, a description of the population from which the groups or cohorts will be selected (e.g., primary care clinic, community sample, residents of a certain town). (Limit: 1000 characters)		NLM

Variable Name	Variable Label	Value List	Entity Name	NLM Required	FDAAA Required	NLM Definitions	CTTI Definitions	Variable Source
study_type	Study Type	Interventional<br>Expanded access<br>Observational	CLINICAL_STUDY	Yes	Yes	Nature of the investigation.<br><br>* Interventional: studies in human beings in which individuals are assigned by an investigator based on a protocol to receive specific interventions. Subjects may receive diagnostic, therapeutic or other types of interventions. The assignment of the intervention may or may not be random. The individuals are then followed and biomedical and/or health outcomes are assessed.<br><br>* Observational: studies in human beings in which biomedical and/or health outcomes are assessed in pre-defined groups of individuals. Subjects in the study may receive diagnostic, therapeutic, or other interventions, but the investigator does not assign specific interventions to the subjects of the study.<br><br>* Expanded Access: records describing the procedure for obtaining an experimental drug or device for patients who are not adequately treated by existing therapy, who do not meet the eligibility criteria for enrollment, or who are otherwise unable to participate in a controlled clinical study. Expanded Access records are used to register all types of non-protocol access to experimental treatments, including protocol exception, single-patient		NLM

Variable Name	Variable Label	Value List	Entity Name	NLM Required	FDAAA Required	NLM Definitions	CTTI Definitions	Variable Source
verification_date	Record Verification Date		CLINICAL_STUDY	Yes	Yes	Date the protocol information was last verified. Verification date is shown along with organization name on ClinicalTrials.gov to indicate to the public whether the information is being kept current, particularly recruiting status and contact information. Update verification date when reviewing the record for accuracy and completeness, even if no other changes are made.		NLM
why_stopped	Why Study Stopped		CLINICAL_STUDY	No	No	For suspended, terminated or withdrawn studies, provide a brief explanation of why the study has been halted or terminated. If desired, use brief summary or detailed description to provide additional information. (Limit: 160 characters)		NLM
mesh_condition_id	Primary Key for CONDITION_BROWSE		CONDITION_BROWSE	N/A	N/A		Numeric sequential ID generated by system. MESH_CONDITION_ID_SEQ	CTTI
mesh_term	Condition MeSH terms		CONDITION_BROWSE	No	No	N/A	Condition MeSH terms generated by NLM algorithm	NLM
nct_id	ClinicalTrials.gov registry number		CONDITION_BROWSE	No	No	N/A	The NCT number is a unique number assigned by clinicaltrials.gov to each clinical trial.	NLM
condition	Condition		CONDITIONS	Yes	Yes	Primary disease or condition being studied, or focus of the study. Diseases or conditions should use the National Library of Medicine's Medical Subject Headings (MeSH) controlled vocabulary when possible.		NLM
condition_id	Primary Key for CONDITIONS		CONDITIONS	N/A	N/A		Numeric sequential ID generated by system. CONDITION_ID_SEQ	CTTI
nct_id	ClinicalTrials.gov registry number		CONDITIONS	No	No	N/A	The NCT number is a unique number assigned by clinicaltrials.gov to each clinical trial.	NLM
design_id	Primary Key for DESIGNS		DESIGNS	N/A	N/A		Numeric sequential ID generated by system. DESIGN_ID_SEQ	CTTI

Variable Name	Variable Label	Value List	Entity Name	NLM Required	FDAAA Required	NLM Definitions	CTTI Definitions	Variable Source
design_name	Design Name (includes Primary purpose, Interventional Model, Masking, Allocation, Study Classification, Observational Study Model, Timeperspective, and Control)	Primary Purpose<br>Control<br>Time Perspective<br>Additional Descriptors<br>Observational Model<br>Endpoint Classification<br>Interventional Model<br>Masking<br>Allocation	DESIGNS	Yes (Interventional Study Design)<br><br>No (Primary Purpose)<br><br>No (Intervention Model)<br><br>No (Allocation)<br><br>No (Study Classification)<br><br>Yes (Observational Study Model)<br><br>Yes (Time Perspective)	Maybe (Interventional Study Design)<br><br>Yes (Primary Purpose)<br><br>Maybe (Intervention Model)<br><br>Maybe (Allocation)<br><br>No (Study Classification)<br><br>No (Observational Study Model)<br><br>No (Time Perspective)	Interventional Study Design * (FDAAA)<br><br>Definition: Primary investigative techniques used in the protocol. Select the most appropriate term describing the protocol from each of the following data elements.<br><br>Primary Purpose FDAAA - reason for the protocol<br><br>o Treatment: protocol designed to evaluate one or more interventions for treating a disease, syndrome or condition<br><br>o Prevention: protocol designed to assess one or more interventions aimed at preventing the development of a specific disease or health condition<br><br>o Diagnostic: protocol designed to evaluate one or more interventions aimed at identifying a disease or health condition<br><br>o Supportive Care: protocol designed to evaluate one or more interventions where the primary intent is to maximize comfort, minimize side effects or mitigate against a decline in the subject's health or function. In general, supportive care interventions are not intended to cure a disease.	Derived from NLM's data element <study_design>	CTTI

Variable Name	Variable Label	Value List	Entity Name	NLM Required	FDAAA Required	NLM Definitions	CTTI Definitions	Variable Source
design_value	Design Value (includes enumerated values for each of the 'Design Name' category)	Psychosocial<br>Health services research<br>Ecologic or community<br>Natural History<br>Defined Population<br>Case-crossover<br>Case-only<br>Case-control<br>Cohort<br>Uncontrolled<br>Placebo Control<br>Historical Control<br>Dose Comparison<br>Active<br>Pharmacokinetics/dynamics Study<br>Pharmacodynamics Study<br>Pharmacokinetics Study<br>Bio-availability Study<br>Bio-equivalence Study<br>Safety/Efficacy Study<br>Efficacy Study<br>Safety Study<br>N/A<br>Random Sample<br>Non-randomized<br>Randomized<br>N/A : single study<br>Double blind (Participant, Investigator, outcome assessor, Caregiver)<br>Single blind (Participant, Investigator,	DESIGNS	Yes (Interventional Study Design)<br><br>No (Primary Purpose)<br><br>No (Intervention Model)<br><br>No (Allocation)<br><br>No (Study Classification)<br><br>Yes (Observational Study Model)<br><br>Yes (Time Perspective)	Maybe (Interventional Study Design)<br><br>Yes (Primary Purpose)<br><br>Maybe (Intervention Model)<br><br>Maybe (Allocation)<br><br>No (Study Classification)<br><br>No (Observational Study Model)<br><br>No (Time Perspective)	Included in "Design name"	Derived from NLM's data element <study_design>	CTTI
masked_role	Masked Role		DESIGNS	No	Maybe	Included in "Design name"<br><br>If Single Blind or Double Blind is selected, check the role(s) that are to be masked: Subject, Caregiver, Investigator or Outcomes	Derived from NLM's data element <study_design>	CTTI

Variable Name	Variable Label	Value List	Entity Name	NLM Required	FDAAA Required	NLM Definitions	CTTI Definitions	Variable Source
nct_id	ClinicalTrials.gov registry number		DESIGNS	No	No	N/A	The NCT number is a unique number assigned by clinicaltrials.gov to each clinical trial.	NLM
facility_id	Primary Key for FACILITIES		FACILITIES	N/A	N/A		Numeric sequential ID generated by system. FACILITY_ID_SEQ. A record was generated in FACILITIES table for every NCT_ID (unique study ID), even when there is no data under address (<name>) for that NCT_ID from ClinicalTrials.gov dataset. In this case the record has NULL value for name variable.	CTTI
location_id	Foreign Key for LOCATIONS		FACILITIES	N/A	N/A		Numeric sequential ID generated by system. LOCATION_ID_SEQ	CTTI
name	Facility Name		FACILITIES	Yes	Maybe	Full name of the organization where the protocol is being conducted. (Limit: 254 characters). Examples: UCLA Eye Institute; Springfield Memorial Hospital		NLM
nct_id	ClinicalTrials.gov registry number		FACILITIES	No	No	N/A	The NCT number is a unique number assigned by clinicaltrials.gov to each clinical trial.	NLM
arm_group_label	Intervention Arms/Groups Label		INTERVENTION_ARM_GROUPS	Yes	Maybe	Arm Label - the short name used to identify the arm. (Limit: 62 characters).<br><br>* (FDAAA)<br><br><br>Group/Cohort Label - the short name used to identify the group. (Limit: 62 characters)<br><br>*		NLM
int_arm_group_id	Primary key for INTERVENTION_ARM_GROUP		INTERVENTION_ARM_GROUPS	N/A	N/A		Numeric sequential ID generated by system. INT_ARM_GROUP_ID_SEQ	CTTI
intervention_id	Foreign Key for INTERVENTIONS		INTERVENTION_ARM_GROUPS	N/A	N/A		Numeric sequential ID generated by system. INTERVENTION_ID_SEQ	CTTI

Variable Name	Variable Label	Value List	Entity Name	NLM Required	FDAAA Required	NLM Definitions	CTTI Definitions	Variable Source
nct_id	ClinicalTrials.gov registry number		INTERVENTION_ARM_GROUPS	No	No	N/A	The NCT number is a unique number assigned by clinicaltrials.gov to each clinical trial.	NLM
mesh_intervention_id	Primary Key for INTERVENTION_BROWSE		INTERVENTION_BROWSE	N/A	N/A		Numeric sequential ID generated by system. MESH_INTERVENTION_ID_SEQ	CTTI
mesh_term	Intervention MeSH terms		INTERVENTION_BROWSE	No	No	N/A	Intervention MeSH terms generated by NLM algorithm	NLM
nct_id	ClinicalTrials.gov registry number		INTERVENTION_BROWSE	No	No	N/A	The NCT number is a unique number assigned by clinicaltrials.gov to each clinical trial.	NLM
int_other_name_id	Primary Key for INTERVENTION_OTHER_NAMES		INTERVENTION_OTHER_NAMES	N/A	N/A		Numeric sequential ID generated by system. INT_OTHER_NAME_SEQ	CTTI
intervention_id	Foreign Key for INTERVENTIONS		INTERVENTION_OTHER_NAMES	N/A	N/A		Numeric sequential ID generated by system. INTERVENTION_ID_SEQ	CTTI
nct_id	ClinicalTrials.gov registry number		INTERVENTION_OTHER_NAMES	No	No	N/A	The NCT number is a unique number assigned by clinicaltrials.gov to each clinical trial.	NLM
other_name	Other Names		INTERVENTION_OTHER_NAMES	No	No	list other names used to identify the intervention, past or present (e.g., brand name for a drug). These names will be used to improve search results in ClinicalTrials.gov. (Limit: 160 characters per name)		NLM
description	Intervention Description		INTERVENTIONS	No	Maybe	Cover key details of the intervention. Must be sufficiently detailed to distinguish between arms of a study (e.g., comparison of different dosages of drug) and/or among similar interventions (e.g., comparison of multiple implantable cardiac defibrillators). For example, interventions involving drugs may include dosage form, dosage, frequency and duration. (Limit: 1000 characters)		NLM

Variable Name	Variable Label	Value List	Entity Name	NLM Required	FDAAA Required	NLM Definitions	CTTI Definitions	Variable Source
intervention_id	Primary Key for INTERVENTIONS		INTERVENTIONS	N/A	N/A		Numeric sequential ID generated by system. INTERVENTION_ID_SEQ	CTTI
intervention_name	Intervention Name		INTERVENTIONS	Yes	Yes	For drugs use generic name; for other types of interventions provide a brief descriptive name. (Limit: 160 characters)<br><br>For investigational new drugs that do not yet have a generic name, a chemical name, company code or serial number may be used on a temporary basis. As soon as the generic name has been established, update the associated protocol records accordingly.<br><br>For non-drug intervention types, provide an intervention name with sufficient detail so that it can be distinguished from other similar interventions.		NLM
intervention_type	Intervention Type	Drug<br>Procedure<br>Device<br>Biological<br>Radiation<br>Genetic<br>Other<br>Dietary Supplement<br>Behavioral	INTERVENTIONS	Yes	Yes	Type of intervention: Drug (including placebo), Device (including sham), Biological/Vaccine, Procedure/Surgery, Radiation, Behavioral (e.g., Psychotherapy, Lifestyle Counseling), Genetic (including gene transfer, stem cell and recombinant DNA), Dietary Supplement (e.g., vitamins, minerals), Other		NLM
nct_id	ClinicalTrials.gov registry number		INTERVENTIONS	No	No	N/A	The NCT number is a unique number assigned by clinicaltrials.gov to each clinical trial.	NLM
keyword	Keywords		KEYWORDS	No	No	Words or phrases that best describe the protocol. Keywords help users find studies in the database. Use NLM's Medical Subject Heading (MeSH) controlled vocabulary terms where appropriate. Be as specific and precise as possible. Avoid acronyms and		NLM

Variable Name	Variable Label	Value List	Entity Name	NLM Required	FDAAA Required	NLM Definitions	CTTI Definitions	Variable Source
keyword_id	Primary Key for KEYWORDS		KEYWORDS	N/A	N/A		Numeric sequential ID generated by system. KEYWORD_ID_SEQ	CTTI
nct_id	ClinicalTrials.gov registry number		KEYWORDS	No	No	N/A	The NCT number is a unique number assigned by clinicaltrials.gov to each clinical trial.	NLM
description	Description		LINKS	No	No	Title or brief description of the linked page. If the page being linked is the protocol's home page on the sponsor's Web site, include the words "Click here for more information about this study:" and provide the name of the protocol. (Limit: 254 characters)		NLM
link_id	Primary Key for LINKS		LINKS	N/A	N/A		Numeric sequential ID generated by system. LINK_ID_SEQ	CTTI
nct_id	ClinicalTrials.gov registry number		LINKS	No	No	N/A	The NCT number is a unique number assigned by clinicaltrials.gov to each clinical trial.	NLM
url	URL		LINKS	No	No	A Web site directly relevant to the protocol may be entered, if desired. Do not include sites whose primary goal is to advertise or sell commercial products or services. Links to educational, research, government, and other non-profit Web pages are acceptable. All submitted links are subject to review by ClinicalTrials.gov. complete URL, including http:// (Limit: 254 characters)		NLM
degrees	Degree		LOCATION_CONTACTS	No	No	Degree		NLM
email	Email		LOCATION_CONTACTS	Yes	Maybe	Email		NLM
first_name	First Name		LOCATION_CONTACTS	No	No	First Name		NLM

Variable Name	Variable Label	Value List	Entity Name	NLM Required	FDAAA Required	NLM Definitions	CTTI Definitions	Variable Source
full_name	Full Name		LOCATION_CONTACTS	No	No		Full name parsed out of NLM's data element <last_name> which contains concatenated first name, middle initial, last name, and degree. For future implementation.	CTTI
last_name	Last Name		LOCATION_CONTACTS	Yes	Maybe	Last Name	concatenated first name, middle initial, last name, and degree	NLM
location_contact_id	Primary Key for LOCATION_CONTACTS		LOCATION_CONTACTS	N/A	N/A		Numeric sequential ID generated by system. LOCATION_CONTACT_ID_SEQ	CTTI
location_id	Foreign Key for LOCATIONS		LOCATION_CONTACTS	N/A	N/A		Numeric sequential ID generated by system. LOCATION_ID_SEQ. It creates new record in the LOCATIONS table per study even when there is no data for any fields in LOCATIONS table	CTTI
middle_initial	Middle Initial		LOCATION_CONTACTS	No	No	Middle Initial		NLM
nct_id	ClinicalTrials.gov registry number		LOCATION_CONTACTS	No	No	N/A	The NCT number is a unique number assigned by clinicaltrials.gov to each clinical trial.	NLM
person_type	Type of Location contact (contact, backup, or investigator)	Contact<br>Contact Backup<br>Investigator	LOCATION_CONTACTS	Yes	Maybe	Facility Contact: Person to contact where the protocol is being conducted.<br>* (FDAAA)<br>Facility Contact Backup: Person to contact if Facility Contact is not available (i.e., a second contact person).<br>Investigators (at the protocol location)	Indicates whether a person type is <investigator>, <contact>, <contact_backup>	CTTI

Variable Name	Variable Label	Value List	Entity Name	NLM Required	FDAAA Required	NLM Definitions	CTTI Definitions	Variable Source
phone	Phone		LOCATION_CONTACTS	Yes	Maybe	Phone		NLM
phone_ext	Phone extension		LOCATION_CONTACTS	No	No	Phone extention		NLM
role	Location Official's Role (Principal Investigator or Sub Investigator)	Principal Investigator<br>Sub-Investigator	LOCATION_CONTACTS	No	No	Site Principal Investigator or Site Sub-Investigator		NLM
location_id	Primary Key for LOCATIONS		LOCATIONS	N/A	N/A		Numeric sequential ID generated by system. LOCATION_ID_SEQ. A record was generated in LOCATIONS table for every NCT_ID (unique study ID), even when there is no data under address (<status>) for that NCT_ID from ClinicalTrials.gov dataset. In this case the record has NULL value for status variable.	CTTI
nct_id	ClinicalTrials.gov registry number		LOCATIONS	No	No	N/A	The NCT number is a unique number assigned by clinicaltrials.gov to each clinical trial.	NLM
status	Recruitment Status	Not yet recruiting<br>Active not recruiting<br>Withdrawn<br>Terminated<br>Suspended<br>Completed<br>Enrolling by invitation<br>Recruiting	LOCATIONS	Yes	Yes	Protocol accrual activity at a facility.		NLM
mesh_id	MeSH ID or tree number		MESH_TREES	N/A	N/A		MeSH ID or tree number imputed from MeSH term found in a study using MeSH thesaurus.	CTTI
mesh_term	Condition or Intervention MeSH term		MESH_TREES	No	No	N/A	Condition or Intervention MeSH terms generated by NLM Algorithm	NLM

Variable Name	Variable Label	Value List	Entity Name	NLM Required	FDAAA Required	NLM Definitions	CTTI Definitions	Variable Source
mesh_tree_id	Primary Key for MESH_TREES		MESH_TREES	N/A	N/A		Numeric sequential ID generated by system. MESH_TREE_SEQ	CTTI
mesh_type	Interventional or Conditional MeSH term	Interventional Conditional	MESH_TREES	No	No		condition or intervention	CTTI
nct_id	ClinicalTrials.gov registry number		MESH_TREES	No	No	N/A	The NCT number is a unique number assigned by clinicaltrials.gov to each clinical trial.	NLM
node_name	Node Name		MESH_TREES	No	No		Nodes 1 through 12. The node position number in the tree number or MeSH ID. E.g. for MeSH entry 'Head;A01.456', 'A01' is Node 1, and '456' is Node 2.	CTTI
node_value	Node Value		MESH_TREES	No	No		Node value is the three character value for each node. E.g. for MeSH entry 'Head;A01.456', Node 1 value is 'A01' and Node 2 value is '456'.	CTTI
mesh_id	MeSH ID or tree number		MESH_TREES_RAW	N/A	N/A		MeSH ID or tree number associated with a MeSH term	CTTI
mesh_term	Condition or Intervention MeSH term		MESH_TREES_RAW	No	No		Condition or Intervention MeSH term from MeSH thesaurus downloaded in July 2010	CTTI
description	Description (for secondary as well as primary outcomes)		OUTCOMES	No	No	Additional information about the outcome measure, if needed for clarification. (Limit: 600 characters)		NLM
measure	Outcome Measure Title (for secondary as well as primary outcomes)		OUTCOMES	Yes	No	A concise name for the specific measure that will be used to determine the effect of the intervention(s) or, for observational studies, related to core objectives of the study and receiving the most emphasis in assessment. (Limit: 254 characters)		NLM
nct_id	ClinicalTrials.gov registry number		OUTCOMES	No	No	N/A	The NCT number is a unique number assigned by clinicaltrials.gov to each clinical trial.	NLM

Variable Name	Variable Label	Value List	Entity Name	NLM Required	FDAAA Required	NLM Definitions	CTTI Definitions	Variable Source
outcome_id	Primary Key for OUTCOMES		OUTCOMES	N/A	N/A		Numeric sequential ID generated by system. OUTCOME_ID_SEQ	CTTI
safety_issue	Safety Issue? (for secondary as well as primary outcomes)	yes<br>no	OUTCOMES	No	Maybe	Is this outcome measure assessing a safety issue? Select: Yes/No		NLM
time_frame	Time Frame (for secondary as well as primary outcomes)		OUTCOMES	No	Maybe	Time point(s) at which outcome measure is assessed. (Limit: 254 characters)		NLM
type	Primary or Secondary Outcome		OUTCOMES	No	Yes	Primary Outcome Measure: Specific key measurement(s) or observation(s) used to measure the effect of experimental variables in a study, or for observational studies, to describe patterns of diseases or traits or associations with exposures, risk factors or treatment.<br><br>Secondary Outcome Measures: Other key measures that will be used to evaluate the intervention(s) or, for observational studies, that are a focus of the study. Specify Title, Time Frame, Description (if needed) and Safety Issue as described above.		NLM
affiliation	Organizational Affiliation		PERSONS	No	No	Full name of the official's organization. If none, specify Unaffiliated.<br><br>(Limit: 255 characters)		NLM
degrees	Degree		PERSONS	No	No	Degree		NLM
email	Email		PERSONS	Yes	Maybe	Email		NLM
first_name	First Name		PERSONS	No	No	First Name		NLM

Variable Name	Variable Label	Value List	Entity Name	NLM Required	FDAAA Required	NLM Definitions	CTTI Definitions	Variable Source
full_name	Full Name		PERSONS	No	No		Full name parsed out of NLM's data element <last_name> which contains concatenated first name, middle initial, last name, and degree. For future implementation.	CTTI
last_name	Last Name		PERSONS	Yes	Maybe	Last Name	concatenated first name, middle initial, last name, and degree	NLM
middle_initial	Middle Initial		PERSONS	No	No	Middle Initial		NLM
name_title	name and title of responsible party		PERSONS	No	Yes	Name/Official Title of Responsible Party - for either the principal investigator or sponsor contact (Limit: 254 characters)		NLM
nct_id	ClinicalTrials.gov registry number		PERSONS	No	No	N/A	The NCT number is a unique number assigned by clinicaltrials.gov to each clinical trial.	NLM
organization	organization of responsible party		PERSONS	No	Yes	Organization of Responsible Party - the sponsor or the principal investigator's organizational affiliation (Limit: 254		NLM
person_id	Primary Key for Persons		PERSONS	N/A	N/A		Numeric sequential ID. PERSON_ID_SEQ	CTTI

Variable Name	Variable Label	Value List	Entity Name	NLM Required	FDAAA Required	NLM Definitions	CTTI Definitions	Variable Source
person_type	Type of Overall official (Overall Study Officials, Central Contact, Central contact backup, Responsible party)	Overall official<br>Resposible party<br>Overall contact backup<br>Overall Contact	PERSONS	No (Overall Study Officials)<br><br>Yes (Central Contact)<br><br>No (Central Contact Backup)<br><br>No (Responsible Party)	No (Overall Study Officials)<br><br>Maybe (Central Contact)<br><br>No (Central Contact Backup)<br><br>Yes (Responsible Party)	Overall Study Officials: Person(s) responsible for the overall scientific leadership of the protocol, including study principal investigator.<br><br>Central Contact or Overall Contact (or Facility Contact required): Person providing centralized, coordinated recruitment information for the entire study. * (FDAAA)<br><br>Central Contact Backup: Person to contact if Central Contact is not available.<br><br>Responsible Party: As defined in US Public Law 110-85, Title VIII, Section 801, the term "responsible party", with respect to a clinical trial, means<br><br>1. the sponsor of the clinical trial (as defined in 21 CFR 50.3) or<br><br>2. the principal investigator of such clinical trial if so designated by a sponsor, grantee, contractor, or awardee, so long as the	Indicates whether a person type is <overall_official><br><br><overall_Contact><br><br><resposible_party><br><br><overall_contact_backup>	CTTI
phone	Phone		PERSONS	Yes	Maybe	Phone		NLM
phone_ext	Phone extension		PERSONS	No	No	Phone extention		NLM
role	Overall Official's Role (Study Chair, Study Director)	Study Chair<br>Study Director	PERSONS	No	No	Position or function of the official. Select one (Study Chair/Study Director/Study Principal Investigator).		NLM

Variable Name	Variable Label	Value List	Entity Name	NLM Required	FDAAA Required	NLM Definitions	CTTI Definitions	Variable Source
citation	Citation		REFERENCES	No	No	Bibliographic reference in NLM's MEDLINE format (Limit: 2000 characters)		NLM
nct_id	ClinicalTrials.gov registry number		REFERENCES	No	No	N/A	The NCT number is a unique number assigned by clinicaltrials.gov to each clinical trial.	NLM
PMID	MEDLINE Identifier		REFERENCES	No	No	MEDLINE Identifier: unique PubMed Identifier (PMID) for the citation in MEDLINE		NLM
reference_id	Primary Key for REFERENCES		REFERENCES	N/A	N/A		Numeric sequential ID generated by system. REFERENCE_ID_SEQ	CTTI
reference_type	Reference or Results Reference		REFERENCES	No	No	Reference: Citations to publications related to the protocol: background and/or results. Provide either the unique PubMed Identifier (PMID) of an article or enter the full bibliographic citation.<br><br>Results Reference: Indicate if the reference provided reports on results from this clinical research study.	indicates whether a reference type is results reference or study reference	CTTI
nct_id	ClinicalTrials.gov registry number		SECONDARY_IDS	No	No	N/A	The NCT number is a unique number assigned by clinicaltrials.gov to each clinical trial.	NLM
sec_id	Primary Key for SECONDARY_IDs		SECONDARY_IDS	N/A	N/A		Numeric sequential ID generated by system. SEC_ID_SEQ	CTTI
secondary_id	Secondary ids		SECONDARY_IDS	No	Yes	Other identification numbers assigned to the protocol, including unique identifiers from other registries and NIH grant numbers, if applicable. (Limit: 30 characters)		NLM
agency	Sponsor Name		SPONSORS	Yes	Yes	Sponsor Name	Sponsor Name	NLM
agency_class	Agency Class		SPONSORS	No	No	N/A	Agency class is a derived field by NLM that indicates the broad catergory of sponsor	NLM
nct_id	ClinicalTrials.gov registry number		SPONSORS	No	No	N/A	The NCT number is a unique number assigned by clinicaltrials.gov to each clinical trial.	NLM

Variable Name	Variable Label	Value List	Entity Name	NLM Required	FDAAA Required	NLM Definitions	CTTI Definitions	Variable Source
sponsor_id	Primary Key for SPONSORS		SPONSORS	N/A	N/A		Numeric sequential ID generated by system. SPONSOR_ID_SEQ	CTTI
sponsor_type	Lead Sponsor or Collaborators		SPONSORS	Yes	Yes	Lead Sponsor: Name of primary organization that oversees implementation of study and is responsible for data analysis. For applicable clinical trials, sponsor is defined in 21 CFR 50.3. (Limit: 160 characters) * FDAAA<br><br>Examples: National Institute of Allergy and Infectious Diseases, Bristol-Myers Squibb.<br><br>Collaborator: Other organizations (if any) providing support, including funding, design, implementation, data analysis and reporting. The data provider is responsible for confirming all collaborators before listing them. Provide up to 10 full names of collaborating organizations. (Limit: 160 characters per	Indicates whether a sponsor type is lead sponsor or collaborator	CTTI
